# Quantification of Chemical Groups and Quantitative HPLC Fingerprint of *Poria cocos* (Schw.) Wolf

**DOI:** 10.3390/molecules27196383

**Published:** 2022-09-27

**Authors:** Yu Yang, Xing-Lin Huang, Zhong-Min Jiang, Xue-Fang Li, Yan Qi, Jie Yu, Xing-Xin Yang, Mei Zhang

**Affiliations:** Yunnan Key Laboratory of Southern Medicine Utilization, College of Pharmaceutical Science, Yunnan University of Chinese Medicine, Kunming 650500, China

**Keywords:** *Poria cocos* (Schw.) Wolf, UV–vis, HPLC, fingerprint, QAMS

## Abstract

**(1)Objective:** In this study, a quantitative analysis of chemical groups (the triterpenoids, water-soluble polysaccharides, and acidic polysaccharides) and quantitative high liquid performance chromatography (HPLC) fingerprint of *Poria cocos* (Schw.) Wolf (PC) for quality control was developed. **(2)** **Methodology:** First, three main chemical groups, including triterpenoids, water-soluble polysaccharides, and acidic polysaccharides, in 16 batches of PC were evaluated by ultraviolet spectrophotometry. Afterward, the quantitative fingerprint of PC was established, and the alcohol extract of PC was further evaluated. The method involves establishing 16 batches of PC fingerprints by HPLC, evaluating the similarity of different batches of PC, and identifying eight bioactive components, including poricoic acid B (PAB), dehydrotumulosic acid (DTA), poricoic acid A (PAA), polyporenic acid C (PAC), 3-epidehydrotumulosic acid (EA), dehydropachymic acid (DPA), dehydrotrametenolic acid (DTA-1), and dehydroeburicoic acid (DEA), in PC by comparison with the reference substance. Combined with the quantitative analysis of multi-components by a single marker (QAMS), six bioactive ingredients, including PAB, DTA, PAC, EA, DPA, and DEA, in PC from different places were established. In addition, the multivariate statistical analyses, such as principal component analysis and heatmap hierarchical clustering analysis are more intuitive, and the visual analysis strategy was used to evaluate the content of bioactive components in 16 batches of PC. Finally, the analysis strategy of three main chemical groups in PC was combined with the quantitative fingerprint strategy, which reduced the error caused by the single method. **(3)** **Results:** The establishment of a method for the quantification of chemical groups and quantitative HPLC fingerprint of PC was achieved as demonstrated through the quantification of six triterpenes in PC by a single marker. **(4)** **Conclusions:** Through qualitative and quantitative chemical characterization, a multi-directional, simple and efficient routine evaluation method of PC quality was established. The results reveal that this strategy can provide an analytical method for the quality evaluation of PC and other Chinese medicinal materials.

## 1. Introduction

Many pharmacological studies have demonstrated that traditional Chinese medicines (TCMs) are vital in the prevention and treatment of human diseases, given their multi-target feature, such as the dried sclerotium of *Poria cocos* (Schw.) Wolf (PC), which is known as Fuling in TCM. From ancient times until now, PC has not only been used in many TCM prescriptions but also in healthy foods, such as PC cake and tea. Modern pharmacological research indicates that the chemical groups in PC have been widely used in clinical treatments due to their various effects, such as diuretic, anti-oxidant, anti-tumor, anti-inflammatory, anti-bacterial, and anti-aging effects, among others [1,2,3,4]. Triterpenoids (TS), water-soluble polysaccharides (WSP), and acidic polysaccharides (AP), among others, are regarded as the major bioactive components of PC. To date, over 121 TS [5] and 64 polysaccharides [6,7,8] have been isolated from PC.

TCMs have a wide variety of bioactive ingredients. In addition, their different places of origin, processing methods, and specifications of decoction pieces could result in different levels of bioactive ingredients, which affect the quality of the herbs or decoction pieces. At present, the quality evaluation methods of PC are seriously lagging behind their biological activity studies and clinical applications. However, there is no such item of content determination documented in the Chinese Pharmacopoeia (2020 edition) for feasible quality control of PC. Currently, only a few methods have been reported, suggesting HPLC, UPLC, LC-MS, or UPLC-MS as the core techniques, which mostly focus on the qualitative analysis of TS in PC [9,10]. Therefore, it is important to establish a multi-faceted, simple, and efficient method for the routine evaluation of the quality of PC through qualitative and quantitative chemical characterization.

HPLC fingerprint technology can be used to analyze information regarding the type and content distribution of the active and ineffective components in Chinese medicine, which is in line with the characteristics of the integrity and fuzziness of Chinese medicine. At the same time, HPLC fingerprint technology combined with chemical pattern recognition analysis can truly and vividly reflect the quality differences of Chinese medicine, as well as reveal the rules between the complex components, which have been widely used in the quality control of drugs and the screening of differential markers. It was a feasible model to identify the authenticity of Chinese medicine and evaluate the quality consistency and product stability. The heatmap hierarchical clustering analysis is a multivariate analysis technique for classifications based on sample characteristics or often based on the fingerprint to distinguish and identify Chinese medicine and its preparations. Principal component analysis is an unsupervised pattern recognition method that utilizes dimension reduction technology to transform multiple indicators into a few representative comprehensive indicators, which can classify samples simply and intuitively. It has been reported that principal component analysis and heatmap hierarchical clustering analysis have been applied in the determination of raw cocoa, processed chocolate [11], monofloral honey [12], Salvia miltiorrhiza, Salvia yunnanensis [13], thyme [14], and fried foods [15] to obtain inter-sample correlations and different factors. The quantitative analysis of important organic substances in Chinese herbal medicine or food is very important for the control of the quality. However, there are limitations of shortage, expenses, and instability of chemical reference materials. In comparison, QAMS (qualitative and quantitative analysis of multiple components using a single marker) can not only reduce the detection time and experimental cost but can also improve the practicability of the method and more effectively and comprehensively control the quality of herbal medicines or plant products. It has been reported that QAMS has been applied in the determination of Rong’e Yishen oral liquid [16], notoginseng [17], and walnut leaves [18].

In this study, multiple perspectives of analysis for the preliminary evaluation of chemical components differences of PC from different origins were finished. Three types of the major bioactive chemical groups in 16 batches of PC from different places of origin were measured by UV–Vis spectrophotometry. Subsequently, HPLC fingerprint [19,20,21,22,23] was combined with QAMS [16,18,24,25,26] so that the qualitative and quantitative analysis of *Poria cocos* (Schw.) Wolf can be achieved. Furthermore, multivariate statistical models, including fingerprint similarity analysis, principal component analysis, and heatmap hierarchical clustering analysis, were used for the quality assessment of the differences in PC from different places. 

## 2. Results and Discussion

### 2.1. Quantitative Analysis of Chemical Groups and Method Validation

To validate the method, a sample (S10) was randomly selected, and the precision test was done by consecutively analyzing the same sample solution six times. The relative standard deviations (RSD) of TS, WSP, and AP of chemical groups in the precision test were found in the range of 0.11–0.64%. Meanwhile, in the reproducibility test, the RSDs were found in the range of 0.69–1.92%. Furthermore, the stability test was done by analyzing the same sample solution (S10) six times at different time points (0, 5, 10, 15, 20, 35, 45, 60, and 120 min) and at room temperature. The RSDs were found in the range of 0.09–1.92%. The recovery test was done by the standard addition method. The samples were spiked with a known amount of standard solution and analyzed with the established UV–vis spectrophotometry method. Average recoveries were found in the range of 97.30–99.29%. These results indicate a good performance of the method and high reliability of data generated. The results are shown in Table S3 in the Supporting Information.

### 2.2. Determination of TS, WSP, and AP in PC

TS, WSP, and AP are the three major active ingredients in PC. For example, TS has been proven to exert potent pharmacological effects [3,27,28,29,30,31,32,33,34,35,36,37,38], such as anti-inflammatory, anti-tumor, and diuretic effects [6,7,8]. Polysaccharides have liver-protective, immune-boosting, and anti-aging effects [28,39]. In this study, three major chemical groups in PC from 16 places of origin were analyzed by UV spectrophotometry. As listed in Table 1, the total TS content was in the range of 1.47–5.58%, WSP content was in the range of 0.48–2.60%, and AP content was in the range of 50.81–88.78%.

### 2.3. HPLC Fingerprint for Alcohol Extract of PC

#### 2.3.1. HPLC Fingerprint and Method Validation in PC

To validate the method, a sample (S10) was randomly selected. For the precision test, the same sample (S10) solution was analyzed consecutively six times. The precision was found in the range of 1.00–4.61%. Meanwhile, for the reproducibility test, the six independent replicate preparations of the same sample (S10) were used for the analysis, and the RSDs of fifteen common peak areas were found in the range of 1.00–3.79%. For the stability test, the same sample solution was analyzed six times at different time points (0, 2, 4, 8, 12, 16, 20, 24, and 48 h) and room temperature and the RSDs of fifteen common peak areas were found in the range of 1.00–4.73%.

The RSDs of all tests were less than 5%, which indicates that the developed method was of good performance and reliability.

#### 2.3.2. Analysis of HPLC Fingerprint and Similarity for Alcohol Extract of PC

**Figure 1:**
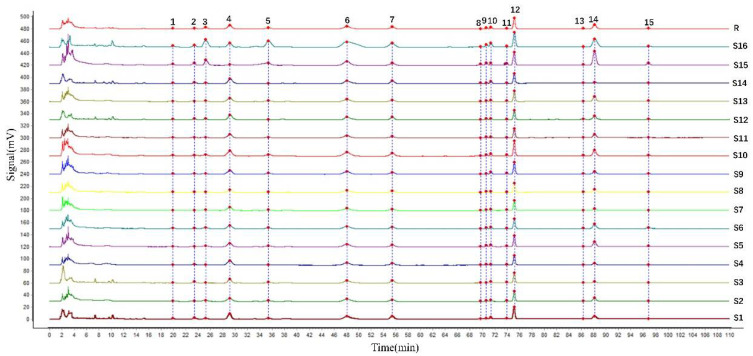
HPLC fingerprints for 16 batches of PC. (R, control chromatogram; S1–S16, 16 batches of PC fingerprints by HPLC).

The HPLC fingerprint of TCM is an effective method to control the quality and reflect detailed chemical information. It could also express its holistic characteristics in a systematic, obvious, and integrated way. The obtained chromatograms of PC from different places of origin were entered into the Similarity Evaluation System for Chromatographic Fingerprint of TCM (version 2012). The chromatogram of S1 was selected as the reference graph. The median method, multi-point calibration, and automatic matching of the peak were used to establish the stacking chromatogram (Figure 1) and control chromatogram (R). Based on the matching information of peaks, 15 peaks were elected as the common peaks. Due to the better peak shape, better separation, and higher response than others, the reference peak of the S10 chromatogram was selected.

The results of the similarity analysis are shown in Table S4 in Supporting Information. The similarity values of the sixteen batches were in the range of 0.880–0.999. Although the above results indicate that the PC fingerprint chromatograms of the sixteen batches demonstrated good similarity, there were still differences in the PC fingerprint chromatograms of the different regions. It also means that PC samples from different places have differences in terms of chemical components. Thus, places of origin could significantly affect the quality of herb; moreover, it has demonstrated that geographical location, climate, and humidity influence the biosynthesis and accumulation of secondary metabolites in organisms. Therefore, we need to determine the concentrations of the characteristic chemical components of PC in fingerprints.

### 2.4. Determination of Six Main Components in PC by QAMS

#### Determination of Six Main Components in an Alcohol Extract of PC and Method Validation

**Figure 2:**
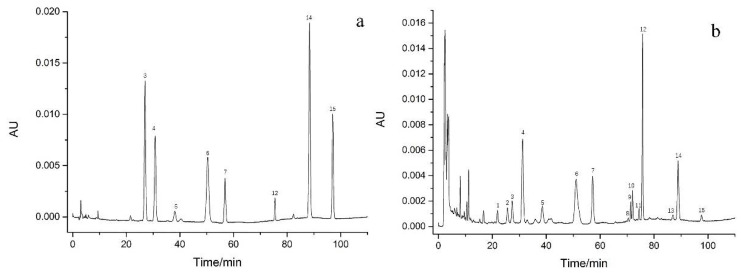
The representative HPLC chromatograms of the standards (**a**) and samples (**b**): 3, poricoic acid B; 4, dehydrotumulosic acid; 5, poricoic acid A; 6, polyporenic acid C; 7, 3-epidehydrotumulosic acid; 12, dehydropachymic acid; 14, dehydrotrametenolic acid; 15, dehydroeburicoic acid. However, the chemical structure of the remaining compounds 1, 2, 8, 9, 10, 11, 13 are uncertain.

**Figure 3:**
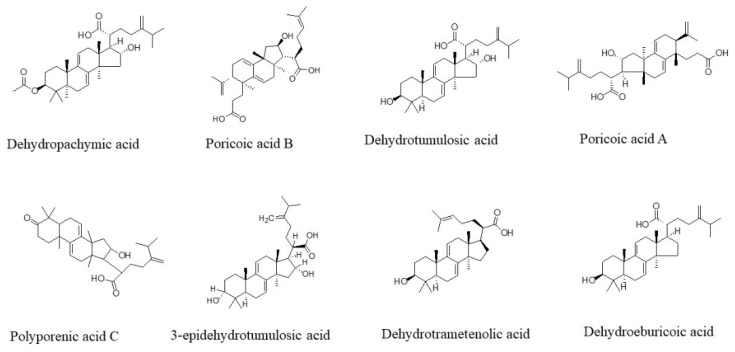
The structure of the eight analytes.

The accurate location of chromatographic peaks in HPLC was essential for the quantitative analysis of samples by the QAMS method. According to the relative retention time (RRT) of the reference chromatogram, the peaks 3, 4, 5, 6, 7, 12, 14, and 15 were recognized as poricoic acid B (PAB), dehydrotumulosic acid (DTA), poricoic acid A (PAA), polyporenic acid C (PAC), 3-epidehydrotumulosic acid (EA), dehydropachymic acid (DPA), dehydrotrametenolic acid (DTA-1), and dehydroeburicoic acid (DEA), respectively (Figure 2). The structures of the eight analytes are shown in Figure 3. However, the RCF values for two (PAA and DTA-1) of these substances did not meet the requirements for the QAMS study. Thus, the remaining six components (PAB, DTA, PAC, EA, DPA, and DEA) were used. EA was selected as the appropriate internal reference for the other five components.

The precision was found in the range of 1.00–3.87%. Meanwhile, for the reproducibility test, the RSDs were found in the range of 1.00–3.79%. The stability test was done by analyzing the sample solution six times at different time points (0, 2, 4, 8, 12, 16, 20, and 24 h) and room temperature, and the RSDs were found in the range of 1.00–3.93%. The recovery test was analyzed by the standard addition method. The samples were spiked with a known amount (high, medium, and low levels) of standard solution, and then analyzed with the established HPLC method. Average recoveries were found in the range of 98.23–105.52%, with RSDs of ˂4.50%. The above results are shown in Table 2. These results indicate the good performance of the HPLC method and the good reliability of its data.

We determined whether QAMS could replace ESM in determining the concentrations of substances in PC. In this study, the concentrations of PAB, DTA, PAC, DPA, and DEA in 16 batches of PC were determined by ESM and QAMs to evaluate the feasibility, respectively, and compare the differences between ESM and QAMS. First, we calculated the ratio of QAMS to ESM content (r Q/E). The calculation results (Table 3) show that the closer r Q/E was to 1.000, the more similar the concentration results of the two methods. Second, we subjected the data to t-test statistical analysis, and the P values were both greater than 0.05. The above results show that there was no significant difference between the two methods. In addition, all samples contained these five analytes; however, the content calculations showed that the concentrations of the same components varied widely between different samples. For example, the concentration of DAB was found in the range of 0.003–0.075 mg/mg. To provide a visual representation of the classification between the different samples, PCA and HCA were used to further analyze these samples.

### 2.5. Principal Component Analysis

PCA was performed using IBM SPSS Statistics for Windows, version 26 (IBM Corp., Armonk, NY, USA). The peak areas of 15 common peaks in HPLC fingerprint were subjected to PCA. The characteristic value and contribution rates are shown in Table S7 in Supporting Information. PCA effectively displayed the differences between the 16 batches of PC. The first three principal components (P1–3) had an Eigenvalue greater than 1, and their cumulative variance contribution rate was 85.441%. This means that the obtained three principal components could be used to represent most of the information of the original 15 common peaks. Therein, P1, P2, and P3 accounted for 52.723%, 22.061%, and 10.658% of the total contribution rate, respectively. The Factor load matrix reflects the contribution and action direction of each variable to the principal component. It is listed in Table S8 in Supporting Information that common peaks F3, F5, F6, F7, F10, and F12 contributed significantly to P1; common peaks F1, F8, F11, and F15 contributed more to P2, and common peaks F4 contributed more to P3. The peaks that contributed more were positively correlated with P1, P2, and P3. These PCA results showed that the quality difference of 16 batches of PC was not the contribution of a single component but the interaction of multiple components.

### 2.6. Heatmap Hierarchical Clustering Analysis

**Figure 4:**
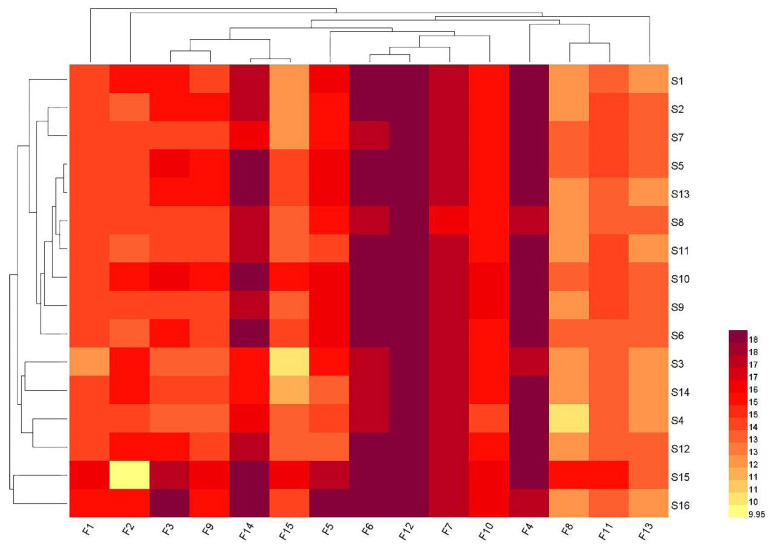
The heatmap HCA for 16 batches of PC.

The principle of the heat map is based on the aggregation of a large amount of experimental data to show results intuitively with a progressive color band. Combining heat maps with HCA not only reflects regional variation in the study population through the sparsity and frequency of data but also provides a visual representation of the classification between samples. This analysis was performed by plotting a heat map using the Image GP web mapping tool using the 15 common peak areas of 16 batches of PC as variables, as shown in Figure 4. The figure shows that the 16 batches of PC samples from different places of origin displayed certain clustering characteristics. It can be divided into two categories by places of origin: S15 and S16 are classified into one category, and the remaining samples are placed in another category. The results of the HCA heat map and total TS analysis were verified. The main reason for the difference in the quality of the PC can be seen on the heat map and may be related to the different content of the chemical components. For example, F4, F7, F12, and F13 were higher in S10 but lower in other samples. The same problem exists in samples S15 and S16. F1, F8, F9, F11, F14, and F15 were higher in S15. F3, F5, F6, and F10 were higher in S16. In addition, F4, F5, F6, F7, F12, and F14 (4, DTA; 5, PAA; 6, PAC; 7, EA; 12, DPA; 14, DTA-1) in the 16 batches of PC were high, which can provide a basis for the subsequent quality control of PC and selected as the indicator component to evaluate the overall quality of PC.

## 3. Materials and Methods

### 3.1. Materials and Reagents

High-purity deionized water was purified with a Milli-Q System (Millipore, Bedford, MA, USA). HPLC-grade methanol and acetonitrile were purchased from Merck (Darmstadt, Germany). All other reagents were of analytical grade or higher. Vanillin (≥99%), D-glucose anhydrous (≥99%), and oleanolic acid (≥98%) were procured from Beijing Enokai Technology Co., Ltd. (Beijing, China). Dehydropachymic acid (≥98%), poricoic acid B (≥98%), dehydrotumulosic acid (≥98%), poricoic acid A (≥98%), polyporenic acid C (≥95%), 3-epidehydrotumulosic acid (≥95%), dehydrotrametenolic acid (≥98%), and dehydroeburicoic acid (≥98%) were procured from Jiangsu Aikang Biomedical Research and Development Co. Ltd. (Jiangsu, China). 

### 3.2. Samples and Samples Collection

Sixteen batches of PC (5 kg each) were collected from different areas in China. The samples were authenticated by Professor Xue-Fang Li and deposited in the Key Laboratory of Preventing Metabolic Diseases of TCM, Yunnan University of Chinese Medicine (Kunming, China). Specifications of the samples evaluated in the present study are shown in Table S1 and Figure S1 in Supporting Information. PC decoction pieces were stored in a cool and dry plant specimen room at room temperature before testing. After pulverization and sifting through a 50-mesh sieve, the powder of the samples for the 16 batches of PC from different places of origin was stored in sealed plastic bags for study analysis.

### 3.3. Sample Preparation

Preparation of total TS extract solution [40]. The aforementioned sample powders (5.0 g) were clearly labeled and put into a 150 mL conical flask with a stopper and bottle mouth rough surface, followed by extraction with 100 mL of 75% ethanol for 35 min in an ultrasonic bath. After cooling, the lost weight was supplemented with 75% ethanol. The total triterpenoid extract solutions were stored at 4 °C.

For the preparation of the total WSP extract solution, [41] the aforementioned sample powders (30.0 g) were soaked in 150 mL of distilled water for 1 h. First, reflux extraction was performed for 3 h, followed by filtering and collection of extractive solutions. Afterward, 150 mL of distilled water was added to the residue, followed by heating and reflux-extracting for 2 h, filtering, and collection of extractive solutions. Finally, 150 mL of distilled water was added to the residue, followed by heating and reflux-extracting for 1 h, filtering, and collection of extractive solutions. The effective components in the medicinal materials were fully extracted through repeated reflux extraction three times. After the extract solutions were cooled and filtered, the insoluble impurities were removed. The extract solutions were concentrated under reduced pressure at 60 °C to 50 mL and 100 mL of water was added. All samples were stored at 4 °C until used for UV–vis spectrophotometry analysis.

For the preparation of total AP extract solution, [42] the residue obtained after the extraction of WSP was dried at 60 °C for 5 h in an oven to a constant weight. Then, each sample powder (1.0 g) was extracted with 70-fold 0.5 M sodium hydroxide solution for 1 h. The extract was filtered through a 500 mesh filter cloth, neutralized with 20% hydrochloric acid, and stood overnight. The suspension solution was subjected to centrifugation (4000 rpm, 10 min), and the supernatant was discarded. Subsequently, the gelatinous precipitate was repeatedly washed with distilled water to remove water-soluble impurities and salts. Lastly, the precipitate was dissolved with the above sodium hydroxide solution and transferred to a 100 mL volumetric flask, and stored in the refrigerator until used for UV–vis spectrophotometry analysis.

### 3.4. Preparation of Standard Solution

Oleanolic acid and D-glucose anhydrous of a certain quality were accurately weighed and dissolved in methanol and ultra-pure water, respectively, to prepare reserve solutions with concentrations of 0.1 mg/mL and 0.2 mg/mL, respectively. The prepared solutions were stored at 4 °C.

### 3.5. Establishment of a Method for the Determination of Chemical Groups (TS, WSP, and AP)

#### 3.5.1. Method Validation of UV–Vis Spectrophotometry Analyses

In this study, precision, repeatability, stability, recovery, and linearity of the sample solution were the indices tested to examine the methodology validation of UV–Vis spectrophotometry using the sample solution (S10). The precision was analyzed by six consecutive tests of the same sample solution. The repeatability was determined by detecting data of six sample solutions from the same batch. The stability was evaluated at room temperature using the same sample solution after 0, 5, 10, 15, 20, 35, 45, 60, and 120 min. The recovery was measured by adding the reference solution for each component at 1:1 concentration with a sample of known concentration (*n* = 6). Linearity was determined by taking 0.05, 0.10, 0.20, 0.40, 0.60, 0.80, 1.50, and 2.00 ml of oleanolic acid reserve solution of the test solution under Section 3.4 and measuring the absorbance according to the method for the determination of total TS stated in Section 3.5.2. Similarly, the absorbance of 0.05, 0.10, 0.20, 0.40, 1.00, 1.20, 1.60, and 2.00 mL of D-glucose anhydrous reserve solution of the test solution under Section 3.4 was measured according to the method for the determination of total polysaccharides in Section 3.5.2. Afterward, the standard curve for oleanolic acid and D-glucose anhydrous were drawn, respectively.

#### 3.5.2. Determination Methods for TS, WSP, and AP

For the total TS content [40] 1 mL of total triterpenoid extract solution in Section 3.3 was measured into a 100 mL volumetric flask and diluted to 100 mL with 75% ethanol. Subsequently, 1 mL of the extract solution was accurately moved to a 25 mL volumetric flask, 1 mL of the extract solution was put into a 20 mL test tube, and the solution in the sample was evaporated using heat. The test tube was cooled to room temperature. Following this, the precipitate was added to 0.4 mL of 5% *w/v* vanillin-glacial acetic acid and 1.8 mL perchloric acid and was then uniformly mixed. The test tubes were treated in a water bath at 70 °C for 20 min. Then, the mixture solution was cooled in an ice bath for 10 min, and 5 ml of glacial acetic acid was added. The solution was evenly mixed, and the absorbance was measured at 540 nm using the reagent as a blank. Using oleanolic acid as the standard to draw the standard calibration curve, the linear equation obtained was A_540_ = 36.641C_o_ + 0.0651; (R² = 0.9995); where A_540_ is the absorbance at 540 nm and C_o_ is the TS content in PC.

For the total WSP content [41] 1 mL of the total WSP extract solution in Section 3.3 was measured in a 25 mL volumetric flask and diluted to 25 mL with distilled water. Subsequently, 1 mL of the extract solution was put into a 20 mL test tube, and 2 ml of 5% phenol solution and 6 mL of concentrated sulfuric acid were added accurately. After cooling for 30 min, the absorbance was measured at 490 nm using the reagent as a blank. For the establishment of the standard calibration curve, D-glucose anhydrous was used as the standard, and the linear equation was A_490_ = 35.436C_d_ + 0.0919; (R² = 0.9994); where A_490_ is the absorbance at 490 nm and C_d_ is the WSP content in PC.

For the total AP content [42] 1 mL of the total AP extract solution in Section 3.3 was measured in a 25 mL volumetric flask and diluted to 25 mL with 0.5 M sodium hydroxide solution. Subsequently, 1 mL of the extract solution was put into a 20 mL test tube, and 2 mL of 5% phenol solution and 6 mL of concentrated sulfuric acid were added. After cooling for 30 min, the absorbance was measured at 490 nm using the reagent as a blank. Similarly, the linear equation obtained was A_490_ = 35.436 C_d_ + 0.0919; (R² = 0.9994).

### 3.6. Establishment of HPLC Fingerprinting for the Total TS

#### 3.6.1. Establishment of HPLC Fingerprinting and Method Validation

In this study, the total TS solution under Section 3.3 was used for this analysis. The extract solution was consecutively filtered through a filter paper and 0.22 µm microporous membrane and then stored in the refrigerator until used for HPLC analysis. Precision, repeatability, and stability of the sample solution were the indices tested to examine the methodology validation of HPLC using the sample solution (S10). The precision was analyzed by six consecutive tests of the same sample solution. The repeatability was determined by detecting data of six sample solutions from the same batch. The stability was evaluated at room temperature using the same sample solution after 0, 2, 6, 8, 12, and 24 h.

#### 3.6.2. HPLC Fingerprinting and Similarity Evaluation of Alcohol Extract of PC

Chromatographic separation was performed on a Waters 2695 Series HPLC system. The system was installed with a Supersil AQ–C18 (4.6 mm × 250 mm, 5µm) column and UV-vis detector system. The optimum instrument conditions are as follows: the injection volume, column temperature, flow rate, and detection wavelength were adjusted for 20 µL, 30 °C, 1.0 mL/min, and 243 nm. The eluent of 0.1% phosphoric acid was used as mobile phase A, and acetonitrile was used as mobile phase B. The gradient elution program used for the analysis was set as shown in Table S2 in Supporting Information.

Specialized software was designed to assess and analyze the similarity of the samples. The software Similarity Evaluation System for Chromatographic Fingerprint of TCM (version 2012) was used. Common peaks were selected based on the following: first, more than 80% of the samples have common peaks; second, the selected common peak should have good separation from the adjacent peak; third, the sum of the common peak areas is more than 90% of the total peak area.

### 3.7. QAMS for Alcohol Extract of PC

#### 3.7.1. Preparation of Standard Solution and Sample Preparation

Standard solutions were prepared by dissolving six reference substances (poricoic acid B, dehydrotumulosic acid, polyporenic acid C, 3-epidehydrotumulosic acid, dehydropachymic acid, and dehydroeburicoic acid) in methanol. Standard solutions were stored at 4 °C until used for further analysis. Then, the mixed standard solutions were diluted, and six appropriate concentrations were selected to establish the calibration curve within the range of 0.61–19.46 mg/mL for poricoic acid B, 0.62–36.86 mg/mL for dehydrotumulosic acid, 0.60–38.40 mg/mL for polyporenic acid C, 1.10–36.35 mg/mL for 3-epidehydrotumulosic acid, 0.68–34.00 mg/mL for dehydropachymic acid, and 0.40–10.75 mg/mL for dehydroeburicoic acid. The extract solution obtained under Section 3.3 was subjected to the QAMS analysis.

#### 3.7.2. QAMS Validation, Durability, and System Suitability Test

The QAMS method introduces a new parameter, which is the relative correction factor (RCF). RCFs are unstable parameters that inevitably influence the accuracy of QAMS results. To analyze the effects of different instruments (Waters 2695 Series HPLC system and Agilent 1200 Series HPLC system), analytical columns (Supersil AQ-C18 [4.6 mm × 250 mm, 5 µm]) and Agilent ZORBAX StableBond SB-C18 (4.6 mm × 250 mm, 5 μm) column) were used at specific flow rates (0.9 and 1.0 mL/min) and column temperatures (28, 30, and 35 °C) to determine the stability of RCF. 

The precision, repeatability, stability, recovery, and linearity of the QAMS method were evaluated by the same chromatographic gradient elution program. The precision was analyzed by six consecutive tests of the same sample solution. The repeatability was determined by detecting the data of six sample solutions from the same batch. The stability was evaluated at room temperature and after 0, 2, 6, 8, 12, and 24 h using the same sample. The recovery was measured by adding the reference solution of three different concentrations for each component at 1:0.5, 1:1, and 1:1.5 concentrations with a sample of known concentration (*n* = 6). Furthermore, the linearity, limits of detection (LODs: S/N = 3), and limits of quantification (LOQs: S/N = 10) of the standard solutions were determined using calibration curves. The results are shown in Table S5 in Supporting Information.

#### 3.7.3. Establishment of QAMS

QAMS also refers to the linear range between the chromatographic response and the concentration of the sample in proportion (*f* = *A*/*C*). Using 3-epidehydrotumulosic acid as an internal referring substance, the calculation of the relative conversion factors (RCFs) at six levels was according to Formula (1). Based on the result of the quantification of other components ($C_{m}$) in the samples, calculations could be performed according to Formula (2).

(1) $$f_{xm}=\frac{f_{x}}{f_{m}}=\frac{C_{x}\times A_{m}}{C_{m}\times A_{x}}$$

(2) $$C_{m}=\frac{C_{x}\times A_{m}}{f_{xm}\times A_{x}}$$

The formulas represent the average RCF of each analyte assayed to EA. The results are shown in Table S6 in the Supporting Information and represent the peak areas of the internal referring substance and other analytes, respectively.

Using the same chromatographic gradient elution program, QAMS was set for the simultaneous determination of six TS in PC. First, 3-epidehydrotumulosic acid was selected as the appropriate internal reference for the five TS poricoic acid B, dehydrotumulosic acid, polyporenic acid C, dehydropachymic acid, and dehydroeburicoic acid. It was used to calculate the RCF ($f_{xm}$) between them. Second, the contents of five TS in PC samples from 16 different producing areas were calculated using Formula (2). Third, the difference between the external standard method (ESM) and QAMS was compared to verify the feasibility of the method.

### 3.8. Multivariate Analysis

Heatmap hierarchical clustering analysis and principal component analysis are unsupervised multivariate exploratory analysis methods that can be used to evaluate classification trends among samples. Heatmap hierarchical clustering analysis was performed based on Squared Euclidean Distance to determine the similarity of samples and categorize them. They were visualized in the form of a heat map. Principal component analysis was required to analyze the principal characteristic components with an Eigenvalue > 1. The characteristic values and contribution rates of the principal components, as well as the factor load matrix, were calculated. These analyses were performed using IBM SPSS Statistics for Windows, version 26 (IBM Corp., Armonk, NY, USA).

## 4. Conclusions

In this study, total TS, WSP, and AP were determined using a color-development system of vanillin-glacial acetic acid and phenol-concentrated sulphuric acid. The results showed that the contents of total TS, WSP, and AP were significantly different between the different places of origin. Therefore, the geographical area also has a certain influence on the quality of the herbs.

In addition, a stable and durable HPLC fingerprint of PC samples was established, and the overall pattern information of the HPLC fingerprint was given by multivariate statistical analysis methods, such as HCA heat map and PCA, which can comprehensively reflect the fingerprint characteristics and the chemical profile. In brief, the aforementioned characteristic information can distinguish PC from different places of origin.

Moreover, the research further combines HPLC fingerprint with QAMS, which establishes a quantitative fingerprint method to provide a comprehensive, objective, and quantitative evaluation of the quality of the PC. The results showed that there are differences in the content of the main components in the PC samples from different places of origin, which may be influenced by temperature, soil, humidity, and artificial cultivation techniques, among others. In conclusion, the quality evaluation method established in the present research provides a comprehensive, simple, economical, and scientific strategy for the quality control of TCMs.

## Figures and Tables

**Table 1:** Results of TS, WSP, and AP determination in PC.

NO.	Content (mg/mg) %		
	TS	WSP	AP
S1	1.53%	1.23%	69.86%
S2	2.73%	0.98%	80.65%
S3	3.31%	2.60%	72.40%
S4	2.15%	1.15%	75.44%
S5	4.22%	1.59%	70.24%
S6	4.87%	1.08%	63.51%
S7	3.30%	1.11%	84.46%
S8	2.26%	0.68%	63.63%
S9	2.78%	1.68%	68.46%
S10	3.20%	1.17%	68.71%
S11	1.47%	2.21%	88.78%
S12	3.41%	1.19%	61.48%
S13	2.59%	1.03%	58.43%
S14	1.50%	0.66%	50.81%
S15	5.64%	1.10%	72.65%
S16	5.87%	0.48%	58.43%

Notes: TS—triterpenoids; WSP—water-soluble polysaccharides; AP—acidic polysaccharides.

**Table 2:** Results of the validation procedure of reference substances (*n* = 6).

Analytes	Regression Equation	R^2^	Linear Range (µg/mL)	LODs (µg/mL^)^	LOQs (µg/mL)	Precision (RSD%, *n* = 6)	Repeatability (RSD%, *n* = 6)	Stability (RSD%, *n* = 6)	Recovery (%, *n* = 6)	
									Average	RSD%
PAB	y = 22976x − 10056	0.9986	0.61–19.46	0.44	1.46	2.47	3.18	2.25	101.78	4.08
DTA	y = 25606x − 25520	0.9968	0.62–36.86	0.58	2.30	1.43	1.40	1.76	103.46	1.80
PAC	y = 35737x − 18430	0.9994	0.60–38.40	0.32	2.24	3.87	3.58	3.62	105.52	3.82
EA (Internal reference)	y = 18451x − 15077	0.9973	1.10–36.35	1.10	2.21	1.00	1.00	1.00	100.98	4.30
DPA	y = 28364x + 16934	0.9993	0.68–34.00	0.34	0.68	3.31	3.79	3.39	98.23	4.50
DEA	y = 29463x − 384.58	0.9996	0.40–10.75	0.30	0.74	2.38	1.97	3.93	101.73	2.67

Notes: y, Peak area; x, the concentration of standards (µg/mL). LODs—the limit of detection; LOQs—limit of quantitation.

**Table 3:** Concentrations of six components in 16 batches of PC as determined by the ESM and QAMS method (mg/mg).

Sample	EA	PAB			DTA			PAC			DPA			DEA		
	ESM	ESM	QAMS	r _E/Q_	ESM	QAMS	r _E/Q_	ESM	QAMS	r _E/Q_	ESM	QAMS	r _E/Q_	ESM	QAMS	r _E/Q_
S1	0.233	0.055	0.052	1.051	0.308	0.331	0.931	0.197	0.201	0.980	0.268	0.241	1.110	0.005	0.004	1.041
S2	0.170	0.055	0.052	1.053	0.230	0.241	0.954	0.160	0.161	0.993	0.231	0.209	1.102	0.005	0.005	1.038
S3	0.198	-	-	-	0.222	0.232	0.957	0.150	0.150	0.998	0.217	0.198	1.099	-	-	-
S4	0.168	0.044	0.040	1.104	0.315	0.339	0.930	0.144	0.143	1.001	0.214	0.195	1.098	0.007	0.006	1.022
S5	0.191	0.083	0.083	0.989	0.265	0.281	0.942	0.198	0.202	0.980	0.248	0.224	1.106	0.014	0.014	0.999
S6	0.170	0.061	0.060	1.031	0.241	0.254	0.950	0.172	0.174	0.989	0.215	0.196	1.098	0.021	0.021	0.994
S7	0.161	0.047	0.043	1.087	0.247	0.261	0.947	0.153	0.153	0.997	0.219	0.199	1.099	0.003	0.003	1.062
S8	0.155	0.048	0.044	1.084	0.193	0.198	0.972	0.134	0.134	1.006	0.199	0.182	1.093	0.008	0.008	1.014
S9	0.208	0.051	0.048	1.067	0.273	0.290	0.940	0.204	0.209	0.979	0.266	0.239	1.109	0.010	0.010	1.007
S10	0.276	0.068	0.067	1.014	0.381	0.415	0.919	0.272	0.281	0.966	0.347	0.310	1.121	0.025	0.025	0.992
S11	0.164	0.046	0.042	1.095	0.250	0.264	0.946	0.157	0.158	0.994	0.203	0.186	1.095	0.008	0.008	1.013
S12	0.189	0.046	0.043	1.090	0.257	0.272	0.944	0.157	0.158	0.994	0.199	0.182	1.094	0.010	0.010	1.007
S13	0.170	0.049	0.046	1.075	0.262	0.277	0.943	0.169	0.171	0.989	0.231	0.209	1.102	0.014	0.014	1.000
S14	0.184	0.041	0.037	1.124	0.251	0.266	0.946	0.144	0.144	1.001	0.211	0.192	1.097	-	-	-
S15	0.222	0.135	0.143	0.946	0.356	0.385	0.923	0.251	0.259	0.969	0.250	0.226	1.106	0.074	0.075	0.985
S16	0.255	0.305	0.335	0.911	0.189	0.194	0.974	0.455	0.479	0.951	0.349	0.311	1.121	0.017	0.017	0.996

Notes: ƒ _EA/PAB_: RCF of poricoic acid B; ƒ _EA/DTA:_ RCF of dehydrotumulosic acid; ƒ _EA/PAC:_ RCF of polyporenic acid C; ƒ _EA/DPA:_ RCF of dehydropachymic acid; ƒ _EA/DEA:_ RCF of dehydroeburicoic acid; r _E/Q_ = ESM/ QAMS.3.4.2. Evaluation of QAMS and ESM.

## Data Availability

Not applicable.

## Supplementary Materials

The following supporting information can be downloaded at: https://www.mdpi.com/article/10.3390/molecules27196383/s1, Figure S1: Sample information of different batches of PC.; Table S1: Sample information of different batches of PC; Table S2: Chromatographic gradient program; Table S3: Methodological investigation parameters; Table S4: The similarities analysis results of 16 batches of PC; Table S5: Effects of different HPLC instruments, columns, flow rates and column temperatures on RCF; Table S6: RCF of PAB, DTA, PAC, DPA, and DEA; Table S7: Characteristic value and cumulative contribution principal components; Table S8: Factor load matrix.
