# Supplementary material for: Quantification of Chemical Groups and Quantitative HPLC Fingerprint of Poria cocos (Schw.) Wolf
Source: Molecules. 2022 Sep 27;27(19):6383. doi: 10.3390/molecules27196383 (PMC9572968; doi:10.3390/molecules27196383)
Supplement: Supplementary file 1 [file molecules-27-06383-s001.zip › molecules-1934406-supplementary.pdf]

Support Information

Quantification of chemical groups and quantitative HPLC fingerprint of *Poria cocos* (Schw.) Wolf

Yu Yang [1]‡, Xing-Lin Huang [1]‡, [1] , Zhong-Min Jiang [1] ,Xue-Fang Li[1], Yan Qi [1], Jie Yu [1]*, Xing-Xin Yang [1]*, and Mei Zhang [1] *

College of Pharmaceutical Science, Yunnan Key Laboratory of Southern Medicine Utilization, Yunnan University of Chinese Medicine, Kunming, China

‡ Both authors contributed equally to this work.

***Corresponding author: Mei Zhang. Tel/Fax: +86-871-65933303; Email: meizhang213@163.com. Xing-Xin Yang. Tel/Fax: +86-871-65933303; Email: yxx78945@163.com. Jie Yu. Tel/Fax: +86-871-65933303; Email: cz.yujie@gmail.com.**

Table S 1

Sample information of different batches of PC.

No.	Growth location	Date of collection
S1	Changning, Yunnan	November,2020
S2	Chuxiong, Yunnan	November,2020
S3	Chuxiong, Yunnan	November,2020
S4	Dali, Yunnan	November,2020
S5	Guangxi	November,2020
S6	Guizhou	November,2020
S7	Hunan	November,2020
S8	Huaping, Yunnan	November,2020
S9	Jingdong, Yunnan	November,2020
S10	Jinggu, Yunnan	November,2020
S11	Yongping, Yunnan	November,2020
S12	Yongsheng, Yunnan	November,2020
S13	Zhenyuan, Yunnan	November,2020
S14	Shuangbai, Yunnan	November,2020
S15	Hubei	November,2020
S16	Ninglang, Yunnan	November,2020

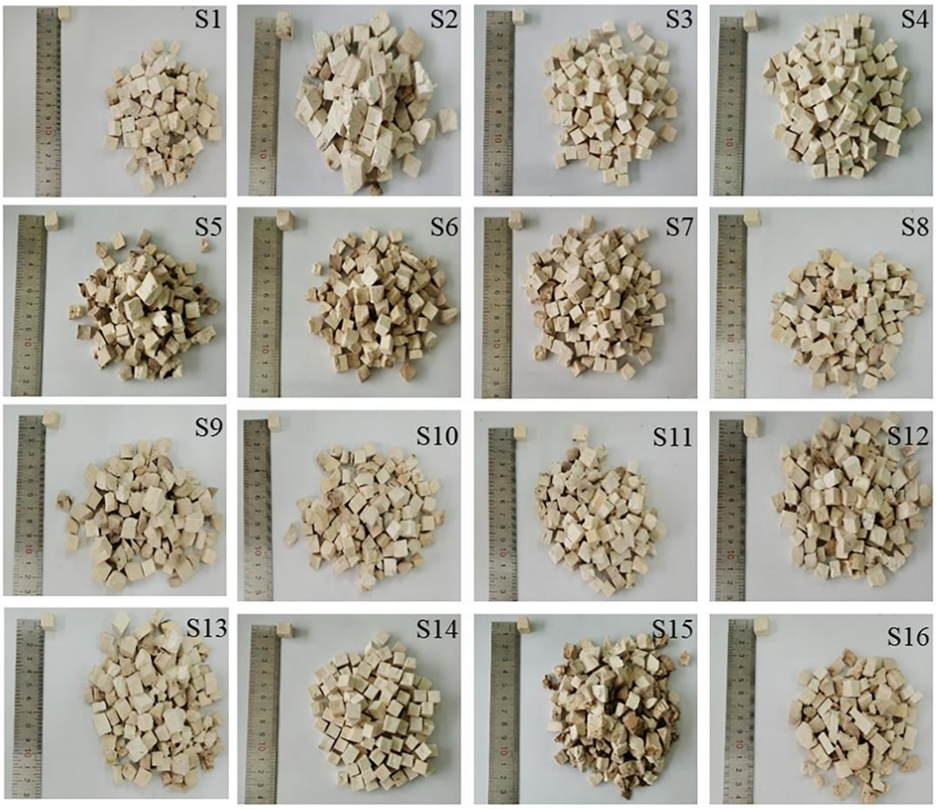

Figure S1. Sample information of different batches of PC.

Table S 2

Chromatographic gradient program.

Time (min)	A (%)	B (%)	Flow rate (mL/min)
0	48	52	1
50	48	52	1
53	42	58	1
60	42	58	1
70	25	75	1
88	25	75	1
95	19	81	1
100	19	81	1

Table S 3

Methodological investigation parameters.

Analyte	Precision (RSD%, n=6)	Repeatability (RSD%, n=6)	Stability (RSD%, n=6)	Recovery (%, n=6)	
				Average	RSD
TS	0.64	1.38	1.05	97.30	1.56
WSP	0.19	1.92	1.92	99.29	2.47
AP	0.11	0.69	0.09	98.05	2.34

Table S 4

The similarities analysis results of 16 batches of PC.

No.	Similarity	No.	Similarity
S1	0.988	S9	0.990
S2	0.995	S10	0.999
S3	0.957	S11	0.991
S4	0.952	S12	0.988
S5	0.996	S13	0.990
S6	0.993	S14	0.953
S7	0.974	S15	0.902
S8	0.993	S16	0.880

Table S 5

Effects of different HPLC instruments，columns，flow rates and column temperatures on RCF.

condition			RCF				
			$f_{EA/PAB}$	$f_{EA/DTA}$	$f_{EA/PAC}$	$f_{EA/DPA}$	$f_{EA/DBA}$
instruments /columns	Waters 2695 Series HPLC system	AQ - C18（4.6 mm × 250 mm, 5μm)	1.232	1.370	1.987	1.840	1.693
		SB - C18（4.6 mm × 250 mm, 5μm)	1.228	1.370	1.987	1.838	1.693
	Agilent 1200 Series HPLC system	AQ - C18（4.6 mm × 250 mm, 5μm)	1.238	1.379	1.959	1.791	1.643
		SB - C18 (4.6 mm × 250 mm, 5μm)	1.238	1.379	1.961	1.773	1.646
flow rates	0.9mL/min	AQ - C18（4.6 mm × 250 mm, 5μm)	1.203	1.352	2.009	2.100	1.752
	1.0mL/min	AQ - C18（4.6 mm × 250 mm, 5μm)	1.202	1.359	2.005	2.007	1.736
column temperature	28℃	AQ - C18（4.6 mm × 250 mm, 5μm)	0.925	1.372	1.983	0.272	2.109
	30℃	AQ - C18（4.6 mm × 250 mm, 5μm)	0.926	1.371	1.996	0.275	2.111
	35℃	AQ - C18（4.6 mm × 250 mm, 5μm)	0.909	1.353	1.966	0.267	2.079

Notes: $f_{EA/PAB}$: RCF of poricoic acid B; $f_{EA/DTA}$: RCF of dehydrotumulosic acid; $f_{EA/PAC}$: RCF of polyporenic acid C; $f_{EA/DPA}$: RCF of dehydropachymic acid; $f_{EA/DEA}$: RCF of dehydroeburicoic acid.

Table S 6

RCF of PAB, DTA, PAC, DPA, and DEA.

No.	$f_{EA/PAB}$	$f_{EA/DTA}$	$f_{EA/PAC}$	$f_{EA/DPA}$	$f_{EA/DEA}$
1	1.237	1.356	1.988	1.859	1.709
2	1.220	1.333	2.017	2.118	1.778
3	1.231	1.337	2.011	2.074	1.769
4	1.232	1.342	2.018	1.969	1.744
5	1.237	1.357	2.013	1.961	1.741
6	1.242	1.355	2.002	1.934	1.732
Mean	1.233	1.347	2.008	1.986	1.746
RSD/%	0.628	0.779	0.570	4.764	1.431

Notes: $f_{EA/PAB}$: RCF of poricoic acid B; $f_{EA/DTA:}$ RCF of dehydrotumulosic acid; $f_{EA/PAC:}$ RCF of polyporenic acid C; $f_{EA/DPA:}$ RCF of dehydropachymic acid; $f_{EA/DEA:}$ RCF of dehydroeburicoic acid.

Table S 7

Characteristic value and cumulative contribution principal components.

Principal component	Characteristic value	Contribution /%	cumulative contribution/%
P1	7.908	52.723	52.723
P2	3.309	22.061	74.783
P3	1.599	10.658	85.441

Table S 8

Factor load matrix.

peaks	Principal component		
	P1	P2	P3
1	0.253	0.933	0.081
2	0.232	-0.724	0.199
3	0.891	0.283	-0.254
4	0.016	0.367	0.768
5	0.929	0.237	-0.198
6	0.974	0.165	-0.020
7	0.813	0.035	0.544
8	0.132	0.902	0.267
9	0.666	0.670	0.110
10	0.843	0.040	0.086
11	0.134	0.837	0.356
12	0.902	0.025	0.348
13	-0.012	0.071	0.693
14	0.592	0.774	0.056
15	0.272	0.903	0.257
